# Mortality of New Orleans, 1844

**Published:** 1844-11-16

**Authors:** 


### MORTALITY OF NEW ORLEANS, 1844.

*Total Number of Deaths of all Diseases.*

From July 1st to 20th, 181; viz. Adults 123, Children under 15 years, 58.

July 20th to August 15th, 237. Adults 151, Children 86, (of Yellow Fever 1.)

August 15th to 31st, 121. Adults 75, Children 46, (of Yellow Fever 4.)

September 1st to 15th, 130. Adults 95, Children 35, (of Yellow Fever 12.)

September 15th to 30th, 157. Adults 111, Children 46, (of Yellow Fever 42.)—*Ibid.*

